# LM-GlycomeAtlas Ver. 1.0: A Novel Visualization Tool for Lectin Microarray-Based Glycomic Profiles of Mouse Tissue Sections

**DOI:** 10.3390/molecules24162962

**Published:** 2019-08-15

**Authors:** Chiaki Nagai-Okatani, Kiyoko F Aoki-Kinoshita, Shuichi Kakuda, Misugi Nagai, Kozue Hagiwara, Katsue Kiyohara, Noriaki Fujita, Yoshinori Suzuki, Takashi Sato, Kiyohiko Angata, Atsushi Kuno

**Affiliations:** 1 Glycoscience and Glycotechnology Research Group, Biotechnology Research Institute for Drug Discovery, National Institute of Advanced Industrial Science and Technology (AIST), Tsukuba, Ibaraki 305-8568, Japan; 2 Glycan & Life Science Integration Center (GaLSIC), Faculty of Science and Engineering, Soka University, Hachioji, Tokyo 192-8577, Japan

**Keywords:** mouse tissue glycome mapping, formalin-fixed paraffin-embedded tissue section, laser microdissection, lectin microarray

## Abstract

For the effective discovery of the biological roles and disease-specific alterations concerning protein glycosylation in tissue samples, it is important to know beforehand the quantitative and qualitative variations of glycan structures expressed in various types of cells, sites, and tissues. To this end, we used laser microdissection-assisted lectin microarray (LMA) to establish a simple and reproducible method for high-throughput and in-depth glycomic profiling of formalin-fixed paraffin-embedded tissue sections. Using this “tissue glycome mapping” approach, we present 234 glycomic profiling data obtained from nine tissue sections (pancreas, heart, lung, thymus, gallbladder, stomach, small intestine, colon, and skin) of two 8-week-old male C57BL/6J mice. We provided this LMA-based dataset in the similar interface as that of GlycomeAtlas, a previously developed tool for mass spectrometry-based tissue glycomic profiling, allowing easy comparison of the two types of data. This online tool, called “LM-GlycomeAtlas”, allows users to visualize the LMA-based tissue glycomic profiling data associated with the sample information as an atlas. Since the present dataset allows the comparison of glycomic profiles, it will facilitate the evaluation of site- and tissue-specific glycosylation patterns. Taking advantage of its extensibility, this tool will continue to be updated with the expansion of deposited data.

## 1. Introduction

Protein glycosylation, one of the most important posttranslational modifications, regulates numerous biological functions by modulating the structure and dynamics of glycan-attached proteins [1]. The glycosylation state varies depending on the type of cells and tissues, and also their physiological state, reflecting the variations and alterations of the glycosylation machinery [1]. Accordingly, the onset and progression of diseases are often accompanied by notable changes in protein glycosylation; hence, disease-related aberrant glycosylation has gathered attention as a reliable target for diagnosis and assessment of the severity of diseases [2,3]. For the effective discovery of glyco-biomarkers exhibiting disease-related glycosylation changes, it is important to directly analyze diseased tissue specimens. This is because body fluids, such as serum, are composed of highly complicated mixtures of glycoproteins derived from various tissues, in which a potential glyco-biomarker protein is an extremely minor component [2,4].

Lectin microarray (LMA) is a lectin-assisted glycan analysis technology used to obtain the global glycomic profiles of both *N*- and *O*-glycans in glycoprotein samples. The profiling, which is based on the patterns of lectin-carbohydrate interactions, allows a highly sensitive and high-throughput analysis using a simple and rapid procedure [4,5,6]. Thus, this technology has complementary roles in assessing the glycomes of glycoprotein samples to those of mass spectrometry (MS)-based approaches, which typically involves complicated sample preparation procedures and analytical conditions optimized for target samples and the type of target glycan (i.e., either *N*- or *O*-glycans). To meet the demands of spatial tissue glycomics, we developed an LMA procedure for the analysis of formalin-fixed paraffin-embedded (FFPE) tissue sections [7], a general type of pathological specimen. Furthermore, we improved the accuracy and reproducibility of this LMA analysis by the additional use of laser microdissection (LMD) for tissue dissection. This standardized LMD–LMA method enabled us to obtain information on site- and tissue-specific protein glycosylation, which was demonstrated by the differential glycomic profiling of five mouse organs (brain, liver, kidney, spleen, and testis) [8].

For detailed analyses of the biological roles and disease-related alterations of protein glycosylation using tissue specimens, it is fundamental and valuable to characterize in advance the glycan structures in the glycoproteome of target cells, sites, and tissues with normal physiology. Thus, to construct our LMA-based tissue glycome mapping atlas, we aimed to collect glycomic profiling data using FFPE sections of mouse tissues by taking advantage of the standardized LMD–LMA method. This project started under the direction of the Biology/Disease-driven Glycoproteome Project (B/D-GPP) of the Human Proteome Organization (HUPO) [4], and is ongoing as a part of a database construction by the Asian Community of Glycoscience and Glycotechnology (ACGG) under the GlyCosmos Project.

Here, as the first atlas of LMA-based tissue glycome mapping data, we developed a novel tool called “LM-GlycomeAtlas” (https://glycosmos.gitlab.io/lm-glycomeatlas). This freely available online tool has a similar interface as GlycomeAtlas (https://glycosmos.gitlab.io/glycomeatlas), which was previously a Flash-based online tool that could visualize glycan structures across tissues based on the MS data provided by the Consortium for Functional Glycomics (CFG) [9]. It has since been updated to use HTML5 so that it is compatible with current web browser technology. Similar to the GlycomeAtlas, the LM-GlycomeAtlas allows users to visualize LMA-based tissue glycomic profiling data that are associated with sample information, including images of dissected areas and the condition of LMA analysis. Using this tool, we present an unpublished dataset consisting of the glycomic profiles of nine mouse tissues (pancreas, heart, lung, thymus, gallbladder, stomach, small intestine, colon, and skin) obtained from two 8-week-old male C57BL/6J mice.

## 2. Results

### 2.1. Collection of Tissue Glycomic Profiling Data

**Figure 1:**
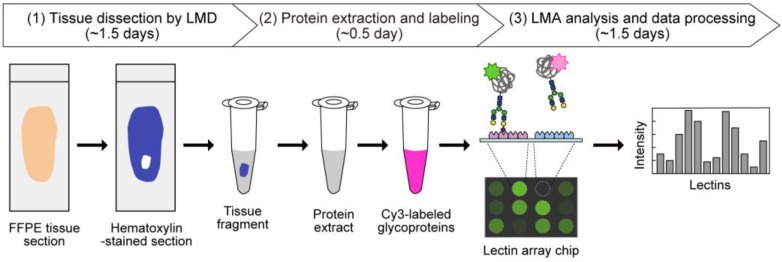
Schematic overview of tissue glycomic profiling by the LMD–LMA method. This method consists of three main steps: (1) tissue dissection from hematoxylin-stained FFPE tissue sections by LMD, (2) extraction and fluorescent labeling of protein samples, and (3) LMA analysis and data processing to obtain glycomic profiles.

In the present dataset, the glycomic profiling data were obtained by a unified procedure (Figure 1). Briefly, 5 μm-thick sections prepared from 4% paraformaldehyde-fixed FFPE tissues were deparaffinized and rehydrated. Note that all procedures for fixation, paraffinization, deparaffinization, and rehydration were the same for all tissues, because these conditions may influence the resulting glycomic profiles. Then, tissue fragments were dissected through the morphological observation of hematoxylin-stained tissue sections using LMD. They were then used for protein extraction using Nonidet P-40 detergent. The resulting protein mixtures were fluorescently labeled with Cy3-succinimidyl ester (Cy3-SE) and analyzed by LMA. In this analysis, the interactions of glycoprotein samples with 45 lectins immobilized on a lectin array chip (Table S1) were quantified based on their fluorescence intensities. The resulting glycomic profile was presented as normalized intensities.

The dataset consisted of the glycomic profiling data of a total of 234 tissue fragments obtained from FFPE sections of nine tissues from two 8-week-old male C57BL/6J mice (Table 1). Three samples obtained from all regions and subregions of the indicated areas of each mouse (i.e., a total of six samples) were analyzed. For several regions, such as the islets of Langerhans, tissue fragments in similar regions on multiple serial sections were collected as individual samples, due to their limited area on one section. Since glycomic profiling data of the tissue samples prepared by the unified procedure were obtained by LMA analyses using lectin array chips of the same lot, all the glycomic profiles can be compared with each other, allowing the evaluation of site- and tissue-specific glycosylation patterns.

### 2.2. Visualization of Tissue Glycomic Profiles

**Figure 2:**
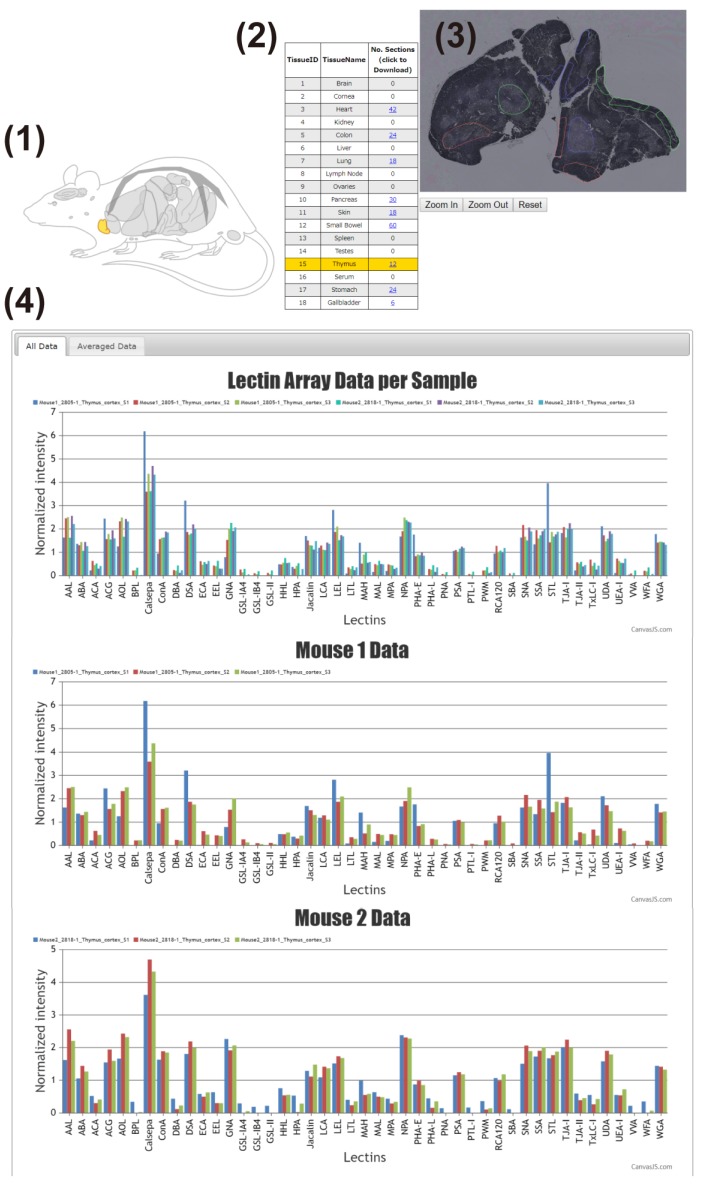
The main interface of the LM-GlycomeAtlas. The display image for the thymic cortex is shown. The main display consists of four main contents: (**1**) mouse whole-body diagram, (**2**) list of tissues, (**3**) representative image of the whole section, and (**4**) graphs of the glycomic profiles.

**Figure 3:**
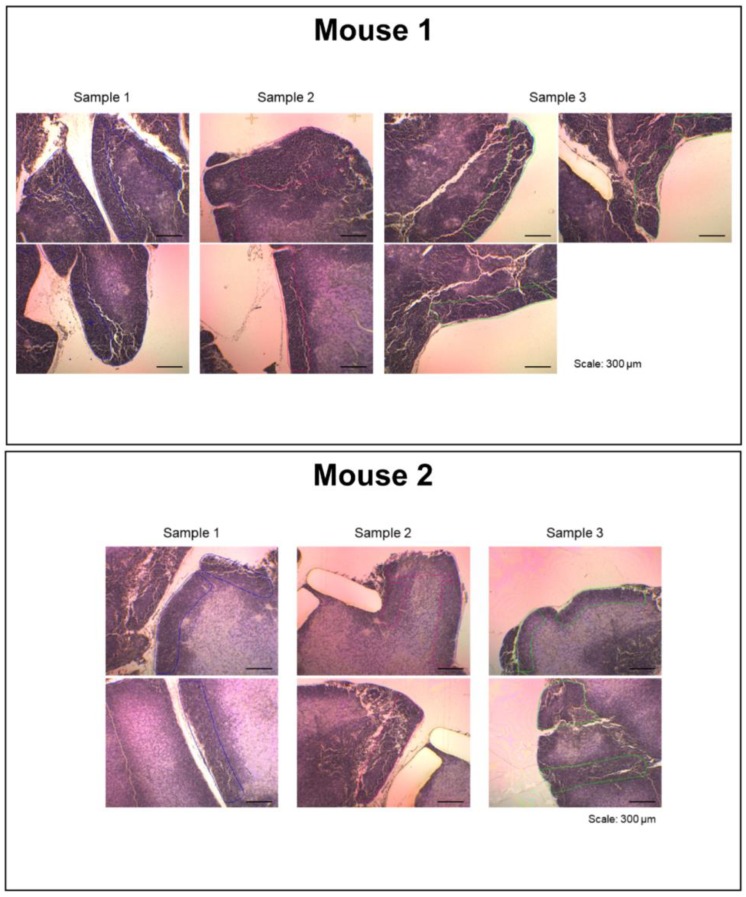
Tissue dissection images shown in a separate window. The display image for the thymic cortex is shown. When the region of interest within the whole section image in the main window (Figure 2) is selected, the tissue dissection images are displayed.

The main interface of the LM-GlycomeAtlas is depicted in Figure 2. The initial screen displays a diagram of mouse whole-body organs and a list including the tissue names and number of data per tissue, allowing users to select the tissue of interest by clicking on the diagram or list. Once a tissue is selected, the scalable whole section image is displayed on the right side of the screen, in which dissected regions are surrounded by frames. Once the region of interest is selected by clicking on it, the images of tissue dissection for two mice are displayed in another window (Figure 3), along with the display of the glycomic profiles of the selected regions at the bottom of the main screen. The display of graphs for three individual samples and averages can be reversibly switched using the “All data” and “Averaged data” tabs, respectively.

The glycomic profiling data, including sample information, can be downloaded as an Excel format file for each tissue by clicking on the links within the tissue list in the main interface (Figure 2). The data files consist of three sheets named “Whole section info,” “Sample info,” and “Lectin array data,” which include sample information summarized in Table 2.

### 2.3. Usage Example of the Dataset: Evaluation of Site-Specific Glycosylation

**Figure 4:**
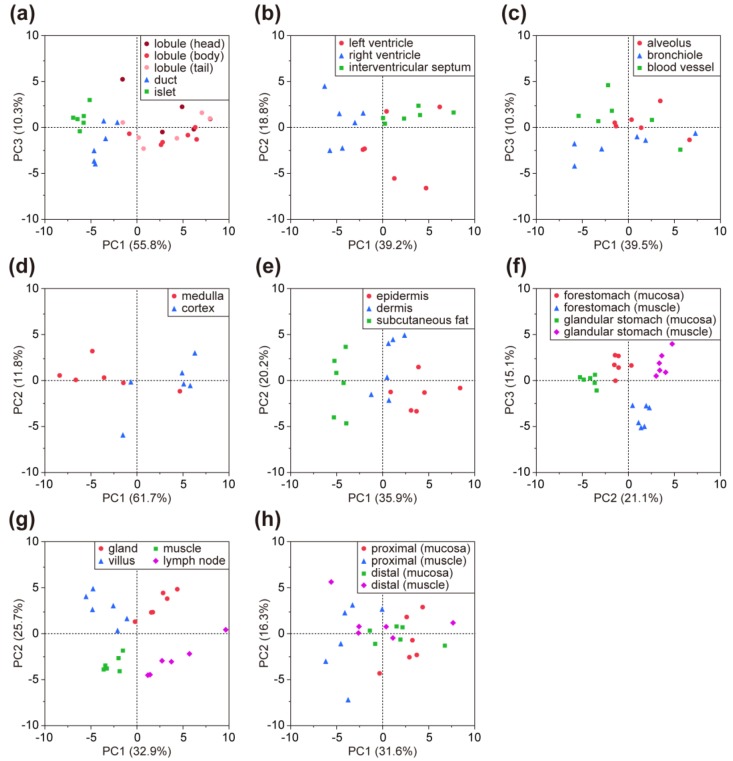
PCA score plots for eight tissues: (**a**) pancreas, (**b**) heart (transverse section), (**c**) lung, (**d**) thymus, (**e**) skin, (**f**) stomach, (**g**) small intestine (ileum), and (**h**) colon. The region/sub-region names and number of the data correspond to those in the sample lists shown in Table 1.

Using the LMA-based glycomic profiling data in the downloaded file (Table 2), users can compare the glycomic profiles consisting of 45 lectins and the mean-normalized values of individual lectins between sites and tissues off-line. Since two or more regions were analyzed for all the tissues, except for the gallbladder (Table 1), the usefulness of the present glycomic profiling dataset was evaluated by comparing multivariable profiles of samples within the same tissue by principal component analysis (PCA). In the PCA score plots for the eight tissues, the plots for the samples appeared to form groups in accordance with the dissected regions (Figure 4), indicating the presence of site-specific glycomic profiling patterns, as expected. Thus, the present dataset is applicable for evaluation of differences in the glycomic profiles between sites and tissues.

## 3. Discussion

Here, we developed the LM-GlycomeAtlas to visualize LMA-based glycomic profiling data obtained from FFPE sections of mouse tissues. The LM-GlycomeAtlas is the first database for LMA-based glycan data analysis, which is highlighted by the fact that many databases for MS-based data of glycan structures and glycomic profiles are currently available [10,11]. This tool is freely available under “Resources” → “Database: Glycomes” → “LM-GlycomeAtlas” in the GlyCosmos Portal (https://glycosmos.org), which is the official web portal of the Japanese Society for Carbohydrate Research. Both the GlycomeAtlas and LM-GlycomeAtlas provide similar user interfaces from the same portal, allowing users to easily compare the MS-based and LMA-based tissue glycomic profiling data.

In addition to visualization directly on the LM-GlycomeAtlas web page, the glycomic profiling data for nine mouse FFPE tissues associated with sample information can also be downloaded from the website in an Excel format file, allowing the use of the data off-line. Although the labeling efficiency depends on the number of the targeting functional groups (i.e., primary amines), the LMA-based glycomic profiles provide meaningful information on the characteristics in the glycan structures attached on glycoproteins. In the present LMD–LMA procedure (Figure 1), the preparation condition of the FFPE tissue sections may influence the resulting glycomic profiles; for example, the tertiary structure of tissue proteins, which determines accessible functional groups and glycans, varies depending on the conditions of tissue fixation (i.e., the type of fixative and incubation time). In addition, the conditions of the heat denaturation of the tissue fragments (i.e., the pH of the buffer and incubation time) have a potential impact on the sialylation status of the glycans, reflecting their affinity to sialic acid-binding lectins. Since the present dataset of nine mouse tissues was obtained using the unified procedure for all tissues (Table 2), all tissue glycomic profiles are comparable with each other. By comparing the glycomic profiles between sites and tissues (Figure 4), a lectin probe can be selected that is suitable for detecting the glycan structures that are relatively abundant in particular sites and tissues. The selected lectin probe can be used for affinity capturing to identify the glycan structures and carrier proteins by MS [12,13,14], and for histochemical analysis to elucidate which cells and sites express glycans of interest [7,8,12,15,16,17,18]. The cell type-specific lectin will facilitate cell type-selective glycomic profiling of FFPE tissue sections as a staining probe, as described previously [19]. In this context, the present tissue glycomic profiling data will contribute to the in-depth analysis of protein glycosylation, facilitating the discovery of disease-related glycosylation alterations.

Since the LM-GlycomeAtlas is a highly extensible application similar to the GlycomeAtlas [9], this atlas can be updated as more data are deposited, including those on mouse tissues other than the present nine tissues as “Ver. 1.0.” In fact, we have collected the glycomic profiling data for five organs, including the brain, liver, kidney, spleen, and testis, of 8-week-old male C57BL/6J mice, in a similar manner as that used for the present nine tissues [8]. The type of tissue sources will also be expanded, including genetically modified and disease model animals, including mice. For example, the glycomic profiles of FFPE heart sections of beta-galactoside alpha-2,3-sialyltransferase 2 (St3gal2)-transgenic mice as a model with dilated cardiomyopathy that were obtained in our recent study [18] may be valuable data. In addition to the deposition of LMA-based data, the integrative addition of other types of data, including MS-based tissue glycome mapping data and histochemical images, will be our main focus in future work. Through the continuous expansion of freely available data and updating for increased user-friendliness, the LM-GlycomeAtlas will be one of the most valuable open resources that contribute to the glycoscience community.

## 4. Materials and Methods

### 4.1. Animals

All animal experiments were performed in accordance with relevant guidelines and regulations and were approved by the Institutional Animal Care and Use Committee at AIST (project code, 2018-082; date of approval, 06/07/2018). C57BL/6J mice were bred and housed in a specific pathogen-free animal facility and had free access to food and water.

### 4.2. Tissue Section Preparation

Tissue sections were prepared as described previously [19]. Briefly, tissues collected from anesthetized 8-week-old male mice were fixed with 4% paraformaldehyde in phosphate buffered saline (PBS) for 24 h at room temperature (RT; approximately 25 °C) and then embedded in paraffin. Tissue sections (5 μm thick) were mounted on poly-L-lysine-coated polyethylene naphthalate- membrane glass slides (Leica Microsystems, Wetzlar, Germany).

### 4.3. Hematoxylin Staining

Tissue section staining was performed as described previously [8,19]. Briefly, tissue sections were deparaffinized three times with xylene for 5 min each, rehydrated in graded concentrations of ethanol, washed with water, and then stained with Mayer’s Hematoxylin Solution (Wako, Osaka, Japan). After each staining process, the sections were washed with Milli-Q water, air-dried at RT, and stored at RT until tissue dissection. Whole section images before and after tissue dissection were obtained using a BZ-X710 microscope (Keyence, Osaka, Japan).

### 4.4. Tissue Dissection and Protein Extraction

Tissue dissection using an LMD7000 LMD system (Leica Microsystems) and subsequent protein extraction were performed as described previously [19]. Briefly, tissue fragments (total area of 0.13–0.91 mm^2^; Table 1) were dissected from hematoxylin-stained sections and collected in a tube. After centrifugation at 20,000× *g* for 1 min at 4 °C, the fragments were heat-denatured in 200 μL of 10 mM citrate buffer, pH 6.0 for 1 h at 95 °C. Note that similar antigen retrieval conditions were also used for MS imaging analyses, where sialic acid-containing *N*-glycans were notably detected [20,21]. After centrifugation at 20,000× *g* for 1 min at 4 °C, 4 μL of a 50% (*w/v*) slurry of microcrystalline cellulose in Dulbecco’s PBS (D-PBS) was added. After discarding 190 μL of the supernatant and adding 190 μL of D-PBS, the fragments were subjected to protein extraction using 20 μL of D-PBS containing 0.5% Nonidet P-40 by sonication. After incubation for 1 h on ice and centrifugation at 20,000× *g* for 1 min at 4 °C, the supernatants were collected as protein extracts.

### 4.5. LMA Analysis

The LMA procedure for protein extracts was performed essentially as described previously [19]. Briefly, 20 μL of protein extracts were fluorescently labeled with Cy3-succinimidyl ester (10 μg protein equivalent; GE Healthcare, Buckinghamshire, UK) for 1 h at RT in the dark. After adjusting the volume to 100 μL with probing buffer (25 mM Tris-HCl, pH 7.5, containing 137 mM NaCl, 2.7 mM KCl, 500 mM glycine, 1 mM CaCl_2_, 1 mM MnCl_2_, and 1% Triton X-100), the sample solutions were incubated for 2 h at RT to quench the excess reagent. After appropriate dilution with the probing buffer when necessary, the sample solutions were added to a well (60 μL/well) on a lectin array chip (LecChip™ ver.1.0; GlycoTechnica, Yokohama, Japan) containing triplicate spots of 45 lectins (Table S1). After incubation overnight at 20 °C and washing three times with the probing buffer, the array chip was scanned using an evanescent-field fluorescence scanner (GlycoStation™ Reader 1200; GlycoTechnica). All data were analyzed using the GlycoStation™ Tools Pro Suite 1.5 software (GlycoTechnica). The net intensity was calculated by subtracting the background from the signal intensity. The data under appropriate gain conditions providing net intensities below 40,000 for all spots were used to obtain glycomic profiles, which are presented as the relative signal intensities of the 45 lectins normalized with the mean value of all lectin signals. The limit of detection calculated as the mean + 3 SD for the NPA signal intensity of the negative control was 0.1 mm^2^ for the analysis of the lobules of the pancreas.

### 4.6. Implementation of LM-GlycomeAtlas

The LM-GlycomeAtlas was implemented by modifying the JavaScript version of GlycomeAtlas [22], which was originally developed in Adobe Flash [9]. Essentially, this tool was built using HTML5, and all data were stored as JSON and SVG images. The complete source code is available at https://gitlab.com/glycosmos/lm-glycomeatlas.

### 4.7. Statistical Analysis

PCA for multivariable glycomic profiling data was performed using the JMP software (version 13.2.1; SAS Institute, Cary, NC, USA).

## Figures and Tables

**Table 1:** Summary of tissue samples in the dataset.

Tissue	Region	Sub-Region	Area (mm^2^)
Pancreas	lobule	head	0.91
		body	0.91
		tail	0.91
	duct		0.91
	islet of Langerhans		0.13–0.84
Gallbladder			0.91
Heart ^1^	left ventricle		0.91
	interventricular septum		0.91
	right ventricle		0.91
Heart ^2^	left atrium		0.50
	right atrium		0.50
	left ventricle		0.50
	right ventricle		0.50
Lung	alveolus		0.91
	bronchiole		0.91
	blood vessel		0.91
Thymus	cortex		0.91
	medulla		0.91
Skin	epidermis		0.91
	dermis		0.91
	subcutaneous fat		0.91
Stomach	forestomach	mucosa	0.91
		muscle	0.91
	glandular stomach	mucosa	0.91
		muscle	0.91
Small Intestine	duodenum	villus	0.91
		gland	0.91
		muscle	0.91
	jejunum	villus	0.91
		gland	0.91
		muscle	0.91
	ileum	villus	0.91
		gland	0.91
		muscle	0.91
		lymph node	0.91
Colon	proximal	mucosa	0.91
		muscle	0.91
	distal	mucosa	0.91
		muscle	0.91

^1^ Transverse section; ^2^ Coronal section.

**Table 2:** Summary of the contents of the Excel data file.

Sheet Name	Contents	Notes
Whole Section Info	Animal (species, strain, age, and sex) and Tissue fixation (buffer and condition)	This sheet is the same for all tissues.
Sample Info	Sample name, Dissected area, Duration, Analyzed sample, LecChip Lot No., Exposure, and Gain	“Duration” means the period from staining until analysis. “Analyzed sample” means the dissected area corresponding to the amount of the sample used for analysis.
Lectin Array Data	Sample name, Lectin name, and Value	This sheet is the original data used for the graphs shown in the LM-GlycomeAtlas.

## Supplementary Materials

The following are available online, Table S1: Abbreviations and carbohydrate specificities of 45 lectins on LecChip.
