# Supplementary material for: LM-GlycomeAtlas Ver. 1.0: A Novel Visualization Tool for Lectin Microarray-Based Glycomic Profiles of Mouse Tissue Sections
Source: Molecules. 2019 Aug 15;24(16):2962. doi: 10.3390/molecules24162962 (PMC6719194; doi:10.3390/molecules24162962)
Supplement: Supplementary file 1 [file molecules-24-02962-s001.pdf]

Table S1. Abbreviations and carbohydrate specificities of 45 lectins on LecChip

	Lectin	Origin	Binding specificity
1	AAL	*Aleuria aurantia*	Terminal α-Fuc, Sia-Le^x and Le^x
2	ABA	*Agaricus bisporus*	Galβ1-3GalNAcα-Thr/Ser (T) and sialyl-T
3	ACA	*Amaranthus caudatus*	Galβ1-3GalNAcα-Thr/Ser (T)
4	ACG	*Agrocybe cylindracea*	Siaα2-3Galβ1-4GlcNAc
5	AOL	*Aspergillus oryzae*	Terminal α-Fuc, Sia-Le^x and Le^x
6	BPL	*Bauhinia purpurea alba*	Galβ1-3GalNAc and NA_3, NA_4
7	Calsepa	*Calystega sepiem*	Man and Maltose
8	ConA	*Canavalia ensiformis*	α-Man (inhibited by presence of bisecting GlcNAc)
9	DBA	*Dolichos biflorus*	GalNAcα-Thr/Ser (Tn) and GalNAcα1-3GalNAc
10	DSA	*Datura stramonium*	(GlcNAc)n, polyLacNAc and LacNAc (NA_3, NA_4)
11	ECA	*Erythrina cristagalli*	Lac/LacNAc
12	EEL	*Euonymus europaeus*	Galα1-3[Fucα1-2Gal] > Galα1-3Gal
13	GNA	*Galanthus nivalis*	Non-substituted α1-6Man
14	GSL-I-A4	*Griffonia simplicifolia*	α-GalNAc and GalNAcα-Thr/Ser (Tn)
15	GSL-I-B4	*Griffonia simplicifolia*	α-Gal
16	GSL-II	*Griffonia simplicifolia*	Agalactosylated *N*-glycan
17	HHL	*Hippeastrum hybrid*	Non-substituted α1-6Man
18	HPA	*Helix pomatia*	Terminal GalNAc
19	Jacalin	*Artocarpus integrifolia*	Galβ1-3GalNAcα-Thr/Ser (T) and GalNAcα-Thr/Ser (Tn)
20	LCA	*Lens culinaris*	Fucα1-6GlcNAc, α-Man and α-Glc
21	LEL	*Lycopersicon esculentum*	(GlcNAc)n and polyLacNAc
22	LTL	*Lotus tetragonolobus*	Fucα1-3GlcNAc, Sia-Le^x and Le^x
23	MAH	*Maackia amurensis*	Siaα2-3Galβ1-3[Siaα2-6GalNAc]α-R
24	MAL-I	*Maackia amurensis*	Siaα2-3Gal
25	MPA	*Maclura pomifera*	Galβ1-3GalNAcα-Thr/Ser (T) and GalNAcα-Thr/Ser (Tn)
26	NPA	*Narcissus pseudonarcissus*	Non-substituted α1-6Man
27	PHA(E)	*Phaseolus vulgaris*	NA_2 and bisecting GlcNAc
28	PHA(L)	*Phaseolus vulgaris*	Tri- and tetra-antennary complex-type *N*-glycan
29	PNA	*Arachis hypogaea*	Galβ1-3GalNAcα-Thr/Ser (T)
30	PSA	*Pisum sativum*	Fucα1-6GlcNAc and α-Man
31	PTL-I	*Psophocarpus tetragonolobus*	α-GalNAc and Gal
32	PWM	*Phytolacca americana*	(GlcNAc)n and polyLacNAc
33	RCA120	*Ricinus communis*	Lac/LacNAc
34	SBA	*Glycine max*	Terminal GalNAc (especially GalNAcα1-3Gal)
35	SNA	*Sambucus nigra*	Siaα2-6Gal/GalNAc
36	SSA	*Sambucus sieboldiana*	Siaα2-6Gal/GalNAc
37	STL	*Solanum tuberosum*	(GlcNAc)n and polyLacNAc
38	TJA-I	*Trichosanthes japonica*	Siaα2-3Galβ1-4GlcNAcβ-R
39	TJA-II	*Trichosanthes japonica*	Fucα1-2Gal, β-GalNAc > NA_3, NA_4
40	TxLC-I	*Tulipa gesneriana*	Man3, bi- and tri-antennary complex-type *N*-glycan, GalNAc
41	UDA	*Urtica dioica*	(GlcNAc)n and polyLacNAc
42	UEA-I	*Ulex europaeus*	Fucα1-2LacNAc
43	VVA	*Vicia villosa*	α-, β-linked terminal GalNAc and GalNAcα-Thr/Ser (Tn)
44	WFA	*Wisteria floribunda*	Terminal GalNAc (e.g., GalNAc β1-4GlcNAc)
45	WGA	*Triticum vulgaris*	(GlcNAc)n and multivalent Sia

Binding specificities are based on Lectin Frontier Database (LfDB; https://acgg.asia/lfdb2).
